# Evidence of Social Understanding Impairment in Patients with Amyotrophic Lateral Sclerosis

**DOI:** 10.1371/journal.pone.0025948

**Published:** 2011-10-05

**Authors:** Marco Cavallo, Mauro Adenzato, Sarah E. MacPherson, Gillian Karwig, Ivan Enrici, Sharon Abrahams

**Affiliations:** 1 Department of Mental Health, “San Luigi Gonzaga” Hospital Medical School, University of Turin, Turin, Italy; 2 Department of Medical Sciences, “Amedeo Avogadro” University of Eastern Piedmont, Novara, Italy; 3 Center for Cognitive Science, Department of Psychology, University of Turin, Turin, Italy; 4 Neuroscience Institute of Turin, Turin, Italy; 5 Human Cognitive Neuroscience-Psychology, University of Edinburgh, Edinburgh, United Kingdom; 6 Centre for Cognitive Aging and Epidemiology, University of Edinburgh, Edinburgh, United Kingdom; 7 Euan MacDonald Centre, University of Edinburgh, Edinburgh, United Kingdom; University of Bologna, Italy

## Abstract

The present study aims at clarifying the nature of the Theory of Mind (ToM) deficits associated with Amyotrophic Lateral Sclerosis (ALS). ToM is the ability to attribute mental states such as intentions and beliefs to others in order to understand and predict their behaviour and to behave accordingly. Several neuroimaging studies reported the prefrontal cortices as the brain region underlying a key ToM ability, i.e. the comprehension of social intentions. Dysfunction of the prefrontal cortices in patients with ALS has been indicated by a range of neuroimaging studies. The frontal syndrome that appears to characterize up to 50% of ALS has been noted to be similar to the profile that characterizes patients with frontotemporal dementia (FTD), a neurodegenerative condition characterised by ToM deficits. In the present paper, we hypothesize that the performance of patients with ALS is significantly worse than healthy controls' performance on tasks requiring the comprehension of social contexts, whereas patients' performance is comparable to healthy controls' performance on tasks not requiring the comprehension of social contexts. To this end, we tested 15 patients with ALS with an experimental protocol that distinguishes between private (non-social) intentions and social intentions. The pattern of results followed the experimental hypothesis: the performance of patients with ALS and healthy controls significantly differed on the comprehension of social context only, with an impairment in patients with ALS. Single case analysis confirmed the findings at an individual level. The present study is the first which has examined and compared the understanding of social and non-social contexts in patients with ALS and shown a specific and selective deficit in the former only. The current findings further support the notion of a continuum of cognitive dysfunction ranging from ALS to FTD, with parallel cognitive profiles in both disorders.

## Introduction

Amyotrophic Lateral Sclerosis (ALS) involves the progressive degeneration of upper and lower motor neurones. ALS has traditionally been considered as a neurodegenerative condition affecting exclusively the motor system, with no repercussions on the cognitive domain. However, numerous studies have now challenged this view, demonstrating the presence of significant cognitive impairment predominantly in the realm of executive functions in a significant proportion of ALS patients, and in language functions in some patients [1]–[5]. In keeping with this, structural and functional neuroimaging has demonstrated that ALS is associated with abnormalities localized mainly in the frontal lobes [6]–[10], and neuropathological investigations have shown the pathological involvement of prefrontal cortices [11]. The frontal syndrome that appears to characterize up to 50% of ALS has been noted to be similar to the profile that characterizes patients with frontotemporal dementia (FTD). Moreover, 5–15% of ALS patients develop a full blown FTD. FTD is the currently preferred term to describe non-Alzheimer type dementia involving mainly the frontotemporal regions of the cerebral cortex [12]–[13]. A link has been established between ALS and FTD on neuropathological [14]–[16], neuroimaging [10], [17] and cognitive [18] grounds. More precisely, it has been proposed that ALS may represent a point on a clinical continuum ranging from ALS, ALS/FTD through to FTD [10], [19].

FTD is characterised by deficits in social cognition and changes in social behaviour, and processes of Theory of Mind (ToM) are now recognised as fundamentally impaired in the disease (for reviews see [20]–[21]). ToM can be defined as the ability to attribute mental states such as intentions and beliefs to others in order to understand and predict their behaviour and to behave accordingly [22]–[23], and it has been recently proposed that the severe social and behavioural problems that often characterize FTD may at least partially be the result of a significant impairment in ToM. This may contribute to patients' difficulty in understanding and managing social interactions appropriately [20], [24].

While there are a significant number of studies on the ToM abilities in FTD, to the best of our knowledge only three studies have directly investigated ToM abilities in patients with ALS [25]–[27]. Gibbons and colleagues [25] used cartoons and stories that require participants to attribute mental states to others, as well as some that pertain to physical events. The analysis of individual patients' results revealed a heterogeneous range of performance ranging from normal to severely impaired. Furthermore, the qualitative analysis of the results showed that the errors made by the patients with ALS were similar to the ones made by patients with FTD [28] although, due to the demanding nature of the tasks used, it is possible that executive dysfunction may have been at the root of the reported deficit. More recently, Girardi et al. [26] described deficits in ALS patients on a test of Judgement of Preference based on eye gaze. This task is a simple undemanding test of ToM and only some of the patients who were impaired on this test also showed evidence of executive dysfunction. The finding that this simple ToM task detected a deficit in more ALS patients than standard tests of executive function suggests an impairment in inferring the mental state of another based on a simple social cue, which is over and above a deficit in executive functions. Furthermore, these authors used the Reading the Mind in the Eyes task, a test consisting in the presentation of photographs of the eye region of human faces, and reported a trend towards significantly lower accuracy scores in ALS patients compared to controls. An altered social awareness and a difficulty in identifying the presence of a faux pas in social situations was reported by a recent study of Meier and colleagues [27]. They used the Faux Pas Test, a test evaluating the affective component of ToM, in which it is essential to understand whether in a social situation somebody said something that they should not have said. Their analysis of individual data revealed that six of 18 patients with ALS were significantly impaired on the faux pas condition and showed a classical dissociation with the control condition.

The present study aims at clarifying the nature of the ToM deficits associated with ALS. We used an experimental protocol deriving from a theoretical taxonomy of intentions proposed in a series of our fMRI studies [29]–[32]. These studies demonstrated that the prefrontal cortices are not necessarily involved in the understanding of other people's intentions *per se*, but primarily in the understanding of the intentions of people who are *currently involved* in social interaction, or who are *preparing for* future social interaction (i.e. when a given social interaction is foreseen, but has not yet occurred). This experimental protocol clearly distinguishes between private (non-social) intentions and social intentions. Private intentions involve the representation of a private goal, i.e. a goal involving only the actor satisfying that particular goal (e.g. working in the kitchen to prepare oneself a meal). Conversely, social intentions involve the representation of a social goal, i.e. a goal of an actor (A) that implies at least another person (B), who is a necessary element for satisfying that goal. In a previous study involving two small groups of FTD and Alzheimer's disease patients [33], we used stories requiring both the comprehension of social contexts and the comprehension of non-social contexts. Interestingly, patients' performance on the social stories was significantly worse than their performance on non-social stories.

As the prefrontal cortices play a crucial role in both the ALS neuropathology [11] and in the understanding of social interactions (a key ToM ability impaired in FTD, [33]), then one may expect impairment to be present in the realms of social understanding in ALS as well. In the present study we use the same social understanding tasks used in our previous study [33]. We hypothesized that the performance of patients with ALS will be significantly worse than healthy controls' performance on tasks requiring the comprehension of social contexts, whereas patients' performance will be comparable to healthy controls' performance on tasks not requiring the comprehension of social contexts.

## Methods

### 1. Ethics Statement

Informed written consent was obtained from all of the participants or from a patient's caregiver if the patient was unable to write. The study was granted approval by the Lothian NHS Research Ethics Committee and was conducted in accordance with the Declaration of Helsinki.

### 2. Participants

The present study involved 15 patients with ALS and 21 healthy controls. Patients with ALS (11 males and 4 females, age range 27–83, mean 59.07±17.60 years, educational level 14.93±3.75 years, mean duration of illness to time of testing 2.68±1.61 years) were retrospectively recruited through the Western General Hospital of Edinburgh (UK), Department of Clinical Neurosciences. Exclusion criteria were the additional presence of other neurological and/or psychiatric disorders such as traumatic brain injuries, strokes or psychosis, or a positive history of alcohol or drug abuse, as well as the presence of significant sensorial impairments and/or extremely severe communication problems that could seriously compromise both the administration of cognitive tests and the interpretation of the relative results. According to the international published criteria [34]–[35], the patients clinically showed the presence of upper and lower motor neuron signs, and a definite or probable diagnosis of amyotrophic lateral sclerosis. Six patients showed the presence of bulbar signs (i.e. the clinically evident involvement of mouth, tongue and throat), whereas 9 patients showed no presence of these signs at the time of testing. Only one of the former patients showed the presence of bulbar signs at the onset of the disease (bulbar onset), whereas the other patients presented with a limb onset.

Healthy controls (14 males and 7 females, age range 29–77, mean 57.48±12.91 years, educational level 17.02±4.15 years) were recruited from a panel of healthy volunteers held by the Department of Psychology at the University of Edinburgh (UK). None of them were related to the patients with ALS involved in the present study, and through a brief clinical interview based on the one reported by Green [36], it was established that none of them had a positive history of neurological and/or psychiatric disorders, or of alcohol or drug abuse.

### 3. General neuropsychological assessment

All of the participants were administered the Graded Naming test (GNT, [37]) to assess their naming ability. Visuospatial abilities were assessed by three subtests of the Visual Object and Space Perception Battery (VOSP, [38]): ‘Object Decision’, ‘Position Discrimination’, and ‘Number Location’. Executive tasks included both timed and untimed tests. Timed tests encompassed letter (P, R, W) and category (animals) spoken verbal fluency tasks (1 minute), as well as the Hayling Sentence Completion test [39]. As an untimed executive test, participants were finally asked to perform the Brixton Spatial Anticipation test [39]. For the verbal fluency tasks, a *verbal fluency index* was calculated [2], [40], in order to control for variation in motor speed: more precisely, participants were first required to generate as many words as possible according to the specific instructions of the various tasks (word generation condition), and then participants were required to read aloud the same words (word read condition). The verbal fluency index (Vfi) was calculated as follows: Vfi = (Time for Word Generation Condition-Time for Word Copy/Read Condition)/ Total Number of Words Generated.

For all of the timed tests, digital recording and the software Praat [41] or a chronometer were used in order to accurately define the time employed by each participant.

### 4. Neuropsychiatric and functional assessment

#### 4.1. Neuropsychiatric assessment

Emotional disturbances were investigated by administering the *Hospital Anxiety and Depression Scale* (HADS, [42]), a brief self-assessment scale that provides a measure of severity of anxiety and depression, adapted for ALS, with the removal of one statement (“I feel as if I am slowed down”) [2].

#### 4.2. Functional assessment

Two measures were employed to monitor the level of functional abilities in patients with ALS. The *Amyotrophic Lateral Sclerosis Functional Rating Scale–Revised* (ALSFRS-R, [43]) is a validated clinical rating scale widely used to identify and follow over time the progression of patients' functional impairment. In addition, patients' daytime somnolence was specifically assessed by the *Epworth Sleepiness Scale* [44], a phenomenon that may be associated with the presence of breathing disorders, and which may affect cognitive performance [45].

### 5. Theory of Mind tasks

Two ToM tasks were used in the present study. The Reading the Mind in the Eyes task (RME, [46]) consists of the presentation of 36 black and white photographs of the eye region of both male and female human faces. Participants were required to choose which of four words (printed below the pictures), best describes what the person in the photograph is thinking or feeling. A control task [47]–[48], designed to investigate participants' ability to correctly identify human physical attributes such as gender, was undertaken subsequently. Participants provided verbal responses and could take as long as they wanted to respond. The RME is a well used test of ToM, but does not require the comprehension of social situations. If a deficit in ALS is specific to the understanding of social situations then no differences should emerge between the performance of ALS patients' and healthy controls'.

The second ToM task is a story completion task presented in a comic strip form that consists of 36 comic strip stories belonging to two theoretical dimensions derived by Ciaramidaro et al. [29]: *Social Contexts (SC)*, and *Non-Social Contexts (N-SC)*. The SC dimension includes prospective social interaction and communicative interaction, and consists of 18 stories depicting both actions with a social goal performed by a single character where a social interaction is foreseen but has not actually taken place (e.g. a single person preparing a romantic dinner), and actions with a social goal performed by two characters in a communicative interaction (e.g. a person obtaining a glass of water by asking another person to get it for her). The N-SC dimension includes stories in which no social interactions are shown, and consists of 9 stories depicting actions performed by a single character with a private goal outside a social interaction (e.g. a single person changing a broken bulb in order to read a book), and 9 stories depicting physical interactions between objects (e.g. a ball blown by a gust of wind knocking over and breaking a glass of water). According to Ciaramidaro and colleagues [29], the stories belonging to the SC dimension require the attribution of social intentions as they concern a social interaction that occurs at the present time or in the future, whereas the stories belonging to the N-SC dimension do not require the attribution of social intentions as they do not concern a social interactions between characters.

Each story consisted of three consecutive pictures (Development Phase), followed by a choice between four concluding pictures (Response Phase). In the *Development Phase* the first and second pictures established a story setting and introduced the characters or the objects involved, while the third picture represented the social or non-social action. In the *Response Phase*, the correct picture represented a probable and congruent effect resulting from the Development Phase, while the incorrect pictures represented an improbable or incongruent effect. Examples of the stories can be found at the following web address: www.psych.unito.it/csc/pers/adenzato/pdf/neurodegdis.pdf.

The story completion task includes a number of important features. Firstly, the stimuli depict simple, high-frequency actions (e.g. pointing towards an object). Secondly, the three drawings that compose each story in the Development Phase remained in front of the participants, so reducing the memory load and allowing them to go back to the story whenever needed during the Response Phase. Lastly, in administering the task, no explicit instructions to pay attention to the nature of the context (social or non-social) were provided, avoiding any direct reference to the characters' intentions. A validation process that involved 33 university students and 33 older adults, conducted prior to the beginning of the present study, enabled the authors to improve the quality of the drawings and the clarity of the social/non-social contexts depicted.

The stories were displayed on a 15.4” WXGA computer screen using the software Presentation 11.0 (Neurobehavioral Systems, Albany, CA, USA). The seating was arranged so that the participants sat in a comfortable chair approximately 0.5 metres from the screen. The first picture of the Development Phase was displayed alone for four seconds in the upper left corner of the screen. Then, the second picture appeared close to the first one. After four seconds, the third picture of the story appeared in the upper right corner of the screen, close to the other two. Participants were asked to look at each story carefully. After four seconds, four possible completions of the story appeared at the same time for 20 seconds below the story pictures (Response Phase). Participants were then required to choose verbally the most appropriate ending of the story amongst the four alternatives provided as fast as they could, by saying aloud the number (1, 2, 3 or 4) associated with the alternative chosen. Given the clinical target involved in the study (patients affected by motor impairments), this paradigm was chosen in order to reduce as much as possible the involvement of the motor domain: for each story, they were required to say aloud only the number associated with their choice. Both their verbal responses and related reaction times were recorded via a sensitive microphone (headphones and microphone two-in-one headset, GEMBIRD) connected to the notebook. Lastly, a control task was proposed in order to take into account participants' verbal speed of reaction. Eighteen stories randomly chosen were presented again according to the same procedure for six seconds each. However, this time one of the alternatives was completely blank. Participants were instructed to look at the four alternatives provided and to say aloud the number associated with the blank picture as fast as they could.

### 6. Statistical analyses

Graphical and statistical exploration of the data by means of box plots, histograms, Q-Q plots and normality tests indicated a normal distribution for most measures, hence parametric tests were used. Non-parametric analyses were undertaken for the VOSP subtests. Statistical analyses were as follows: firstly, group comparisons between patients with ALS and healthy controls on the background (i.e. neuropsychological and neuropsychiatric measures) and experimental (i.e. ToM tasks) variables of interest were performed using unpaired t-tests, Mann-Whitney U-tests or repeated measures Analysis of Variance (ANOVA), as appropriate. Secondly, in order to detect the possible influence of bulbar signs on cognitive performance [25], [40], [49]–[50], two subgroups of patients with ALS were identified according to the presence of bulbar signs at the time of testing, and the comparisons among patients with bulbar signs, patients without bulbar signs and healthy controls were performed using one-way and repeated measures ANOVAs, as appropriate. Thirdly, comparisons of individual patients' and healthy controls' scores on the background neuropsychological and ToM measures were performed using single case methodology [51]–[53]: more precisely, modified t-tests were used to determine whether each individual's scores were significantly lower than the corresponding healthy control group's scores.

Statistical analyses were performed using SPSS^©^ version 18.0 (Statistical Package for the Social Sciences).

## Results

### 1. ALS patients versus healthy controls

#### 1.1. General neuropsychological assessment

The two groups were well matched for age (t(34) = 0.313, not significant, NS) and level of education (t(34)* = *1.550, NS). Scores were expressed as raw scores, with the exception of the Hayling and Brixton tests, for which scaled scores were used. The comparisons between the performance of the two groups are shown in Table 1. Patients with ALS performed worse than healthy controls on the Verbal Fluency letters (t(34) = 3.079, p<0.01), the Verbal Fluency Index (t(34) = 3.254, p<0.01) and the GNT (t(34) = 4.378, p<0.01). No statistically significant differences were found on the other neuropsychological measures.

**Table 1:** Performance of ALS patients and healthy controls on the background neuropsychological tests.

	ALS patients Mean (SD)	Healthy controls mean (SD)	t-test or Mann-Whitney U
	(n = 15)	(n = 21)	
Verbal Fluency: Letters P, R, W (total)	39.53 (14.08)	52.33 (10.87)	3.079*
Verbal Fluency: Index	4.37 (1.85)	2.85 (0.52)	3.254*
Verbal Fluency: Category (total)	18.80 (5.61)	22.05 (4.10)	NS
GNT (0–30)	21.87 (3.38)	26.10 (2.43)	4.378*
VOSP: “Object Decision” (0–20)	18.00 (1.60)	18.29 (1.76)	NS
VOSP: “Position Discrimination” (0–20)	19.93 (0.26)	19.81 (0.40)	NS
VOSP: “Number Location” (0–10)	9.27 (0.89)	9.62 (0.97)	NS
Hayling: “Overall” (1–10)	5.60 (1.24)	6.05 (1.20)	NS
Hayling: “Sensible Completion” (1–7)	5.60 (1.06)	6.05 (0.50)	NS
Hayling: “Unconnected Completion” (1–8)	5.47 (1.36)	5.76 (0.89)	NS
Hayling: Errors (1–8)	6.13 (1.41)	6.33 (1.83)	NS
Brixton (1–10)	6.87 (2.07)	7.10 (1.84)	NS

GNT = Graded Naming Test; NS = not significant; SD = standard deviation; VOSP = Visual Object and Space Perception battery.

*p<0.01.

#### 1.2. General neuropsychiatric and functional assessment

The ALSFRS-R allows researchers and clinicians to detect the possible presence of limb, bulbar and respiratory dysfunction. The patients' mean score was 31.33±7.31 (range: 17–41), and clinically patients varied significantly regarding their level of functional capacities, with nine of them presenting with upper and lower limbs involvement only whereas six patients presented with bulbar signs at the time of testing. The respiratory subscore of the ALSFRS-R combines three questions on Dyspnea, Orthopnea and Respiratory Efficiency. Each question is rated out of a maximum of 4 (4 being normal function) and hence producing a total maximum of 12. Our total patient group had a mean score of 10.59±1.5 (range: 8–12). Hence although some patients had some symptoms of respiratory compromise, this was not marked in any of the patients tested.

The mean score for the Epworth Sleepiness Scale was 5.09±1.87 (range: 2–8, clinical cut-off: 10), and no one reported a clinically significant level of sleepiness. Regarding the HADS, five patients did not perform the scale due to time constraints. The two subscales measuring anxiety and depression respectively did not show the presence of a significant difference between the two groups (anxiety: t(30) = 0.793, NS; depression: t(30) = 1.354, NS), and no patients showed the presence of clinically significant levels of these emotional disorders (*anxiety:* patients with ALS = 3.91±2.26, healthy controls = 4.67±2.71; borderline range: 8–10; *depression:* patients with ALS = 1.82±2.27, healthy controls = 0.95±1.36; borderline range: 8–10).

#### 1.3. Theory of Mind tasks

RME: The number of correct responses for both the experimental (mental states attribution) and the control (gender attribution) tasks were considered. The range of possible scores varied between 0 and 36. Unpaired t-tests revealed the absence of significant differences on both the experimental (patients with ALS = 25.25±4.89, healthy controls 27.00±4.46: t(34) = 0.659, NS) and the control (patients with ALS = 34.75±1.06, healthy controls 34.95±1.40: t(34) = 0.353, NS) tasks.

**Figure 1:**
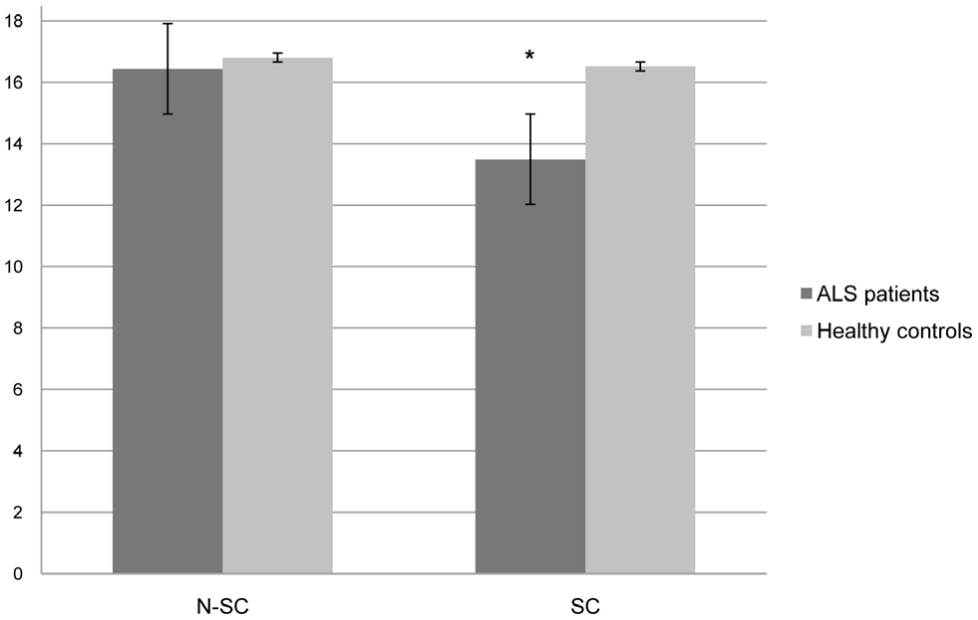
Participants' performance on the story completion task (range of possible values for both the N-SC and SC dimensions: 0–18). N-SC Non-Social items; SC Social items. * p<0.01.

Story Completion Task: Both correct responses and their associated reaction times were analyzed. The number of correct responses for each dimension (SC and N-SC) was considered, and the range of possible scores varied between 0 and 18 for each dimension. A repeated measures ANOVA involving the two groups (patients with ALS and healthy controls) and the two dimensions (N-SC and SC) showed the presence of a statistically significant main effect of group (F(1, 34) = 15.892, p<0.001) and a group x dimension interaction (F(1, 34) = 10.221, p<0.01). Post-hoc paired sample t-tests showed that the performance on the SC dimension was significantly worse in patients with ALS than in healthy controls (t(34) = 3.916, p<0.001), whereas the performance of the two groups on the N-SC dimension did not differ significantly (t(34) 0.813, NS), therefore supporting the experimental hypothesis. Figure 1 shows graphically these comparisons. The comparison in the ALS group of the two types of non-social stories resulted in a statistically significant difference (physical interaction 8.73±0.46, private intention 7.73±1.03, one-sample t-test: 8.489, p<0.001). The comparison in the ALS group of the score on the private intention stories with the averaged score on the social stories resulted in a statistically significant difference (private intention 7.73±1.03; averaged social stories: 6.93±1.37, one-sample t-test: 3.013, p<0.001).

In the analysis of the reaction times, only the reaction times associated with the correct responses were considered. A repeated measures ANOVA involving the two groups (patients with ALS and healthy controls) and the reaction times associated with the two dimensions of interest (SC and N-SC) showed a statistically significant group effect (F(1, 34) 4.876, p<0.05) but not a significant interaction effect (F(1, 34) 0.208, NS), meaning that patients required more time to perform items belonging to both dimensions, compared with healthy controls.

For the control task, all participants scored the maximum of 18 correct responses, thus all reaction times were considered in the following analysis. A t-test performed on the reaction times of the task did not show a significant difference between the two groups (t(34) 0.634, NS), indicating that the ALS patients reported here did not have significantly slowed responding in comparison to controls despite the presence of bulbar dysfunction in some patients.

To conclude, we still investigated possible significant correlations between the scores of the verbal fluency task (letters), the GNT and the story completion task, that were the only tasks that differed across groups. The verbal fluency task and the GNT were significantly correlated to each other (Spearman's *r* = 0.459, p<0.01), whereas no significant correlations were found between the verbal fluency task or the GNT and the story completion task.

### 2. Patients with bulbar signs vs. patients with no bulbar signs vs. healthy controls

#### 2.1. General neuropsychological assessment

The three groups were well matched for age (F(2, 33) 0.546, NS) and level of education (F (2, 33) 2.429, NS). The comparison between the performance of the three groups on the background neuropsychological measures, as well as their demographic data, are shown in Table 2.

**Table 2:** Demographic data of ALS patients with no bulbar signs at the time of testing (i.e. non-bulbar), with bulbar signs at the time of testing (i.e. bulbar) and healthy controls, and relative performance on the background neuropsychological tests.

	Non-bulbar	Bulbar	Healthy controls	F or Kruskal-Wallis H
	(n 9)	(n 6)	(n 21)	
Age in years	62.22 (17.64)	54.33 (18.02)	57.48 (12.91)	NS
Gender (M:F)	7∶2	4∶2	14∶7	-
Education in years	13.67 (3.20)	16.83 (3.96)	17.02 (4.15)	NS
Verbal Fluency: letters P, R, W	40.22 (11.33)	38.50 (18.64)	52.33 (10.87)	4.646*
Verbal Fluency: Index	3.83 (1.27)	5.17 (2.38)	2.85 (0.52)	7.898** §
Verbal Fluency: Category	18.33 (6.60)	19.50 (4.18)	22.05 (4.10)	NS
GNT (0–30)	21.44 (4.07)	22.50 (2.17)	26.10 (2.43)	9.680** ¶
VOSP: “Object Decision” (0–20)	17.44 (1.81)	18.83 (0.75)	18.29 (1.76)	NS
VOSP: “Position Discrimination” (0–20)	20.00 (0.00)	19.83 (0.41)	19.81 (0.40)	NS
VOSP: “Number Location” (0–10)	9.33 (0.71)	9.17 (1.17)	9.62 (0.97)	NS
Hayling: “Overall” (1–10)	5.22 (1.20)	6.17 (1.17)	6.05 (1.20)	NS
Hayling: “Sensible completion” (1–7)	5.33 (1.12)	6.00 (0.89)	6.05 (0.50)	NS
Hayling: “Unconnected completion” (1–8)	5.11 (1.45)	6.00 (1.10)	5.76 (0.89)	NS
Hayling: Errors (1–8)	5.89 (1.69)	6.50 (0.84)	6.33 (1.83)	NS
Brixton (1–10)	5.89 (1.90)	8.33 (1.37)	7.10 (1.84)	NS

GNT Graded Naming Test; NS not significant; SD standard deviation; VOSP Visual Object and Space Perception battery.

*p<0.05.

**p<0.001.

§ Bulbar patients significantly different from healthy controls.

¶ Bulbar and non-bulbar patients significantly different from healthy controls.

#### 2.2. General neuropsychiatric and functional assessment

On the ALSFRS-R, the difference between the two subgroups was statistically significant (bulbar patients 26.67±8.50, non-bulbar patients 34.44±4.59, t(13) 2.312, p<0.05), with bulbar patients showing a higher degree of functional impairment. On the Epworth Sleepiness Scale the two subgroups of patients did not show any significant difference (bulbar patients 5.00±1.00, non-bulbar patients 5.12±2.17, t(13) 0.094, NS). Lastly, the two subscales from the HADS measuring anxiety (bulbar patients 2.67±2.31, non-bulbar patients 4.37±2.20, healthy controls 4.67±2.71) and depression (bulbar patients 1.67±2.08, non-bulbar patients 1.87±2.47, healthy controls 0.95±1.36) did not show the presence of a significant difference between the three groups (anxiety: F(2, 29) 0.797, NS; depression: F(2, 29) 0.902, NS).

#### 2.3. Theory of Mind tasks

RME: There were no significant differences between the three groups for the experimental (F(2, 33) 0.556, NS) or control (F(2, 33) 0.181, NS) RME tasks.

Story Completion Task: A repeated measures ANOVA involving the three groups (bulbar patients, non-bulbar patients and healthy controls) and the two dimensions (N-SC and SC) showed the presence of a statistically significant main effect of group (F(2, 34) 19.353, p<0.001) and a group x dimension interaction (F(2, 34) 4.974, p<0.05). Post-hoc paired sample t-tests showed that the performance of both groups of patients on the SC dimension was significantly worse than in healthy controls (bulbar patients versus healthy controls: t(25) 3.084, p<0.01; non-bulbar patients versus healthy controls: t(28) 2.851, p<0.05), whereas the performance of the two groups of patients on the N-SC dimension did not differ significantly from healthy controls. Table 3 shows the comparisons of interest. Regarding their reaction times, a repeated measures ANOVA involving the three groups (bulbar patients, non-bulbar patients and healthy controls) and the two dimensions (N-SC and SC) did not show the presence of statistically significant group (F(2, 34) 1.559, NS) or interaction (F(2, 34) 0.615, NS) effects.

**Table 3:** Performance on the ToM tasks (scores as correct responses).

	Non-bulbar	Bulbar	Healthy controls	F
	(n 9)	(n 6)	(n 21)	
RME experimental (0-36)	25.33 (3.46)	27.17 (4.26)	27.00 (4.46)	NS
N-SC (0-18)	16.89 (0.78)	15.83 (1.72)	16.81 (1.21)	NS
SC (0–18)	14.22 (3.07)	13.33 (2.34)	16.52 (1.25)	19.353* ¶

N-SC Non-social context; RME Reading the Mind in the Eyes; SC Social Context.

*p<0.001.

¶ Bulbar and non-bulbar patients significantly different from healthy controls.

### 3. Individual patients versus healthy controls

#### 3.1. General neuropsychological assessment

Comparison of individual patient scores on the background neuropsychological tests with healthy controls' means using a modified t-test [51] showed significantly lower scores on the verbal fluency letters in patients 9 (t(21) 2.996, p<0.01), 6 (t(21) 2.187, p<0.05), 11 (t(21) 2.636, p<0.05), 12 (t(21) 2.726, p<0.05), 14 (t(21) 2.187, p<0.05), and 15 (t(21) 2.187, p<0.05); on the verbal fluency category, in patients 1 (t(21) 2.633, p<0.05), 2 (t(21) 2.633, p<0.05), 9 (t(21) 2.157, p<0.05), and 11 (t(21) 2.157, p<0.05). Furthermore, on the GNT patients 2 (t(21) 3.257, p<0.01), 5 (t(21) 3.257, p<0.01), 7 (t(21) 3.257, p<0.01), 9 (t(21) 2.453, p<0.01), 10 (t(21) 2.855, p<0.01), 11 (t(21) 3.257, p<0.01) and 12 (t(21) 2.453, p<0.01) performed poorly, whereas on the VOSP Object Decision and Position Discrimination subtests, patients' performance was equivalent to healthy controls' performance. On the VOSP Number Location”, only patient 8 performed significantly worse than controls (t(21) 2.639, p<0.05). Significant differences were identified on the Hayling “overall” measure in patient 2 (t(21) 2.483, p<0.05); on the Hayling “sensible completion”, in patients 5 (t(21) 4.006, p<0.01), and 10 (t(21) 5.960, p<0.001); on the Hayling “unconnected completion”, in patient 1 (t(21) 4.128, p<0.01); on the Hayling “errors”, in patient 2 (t(21) 2.312, p<0.05), and on the Brixton test, in patient 6 (t(21) 2.708, p<0.05).

#### 3.2. Theory of Mind tasks

**Figure 2:**
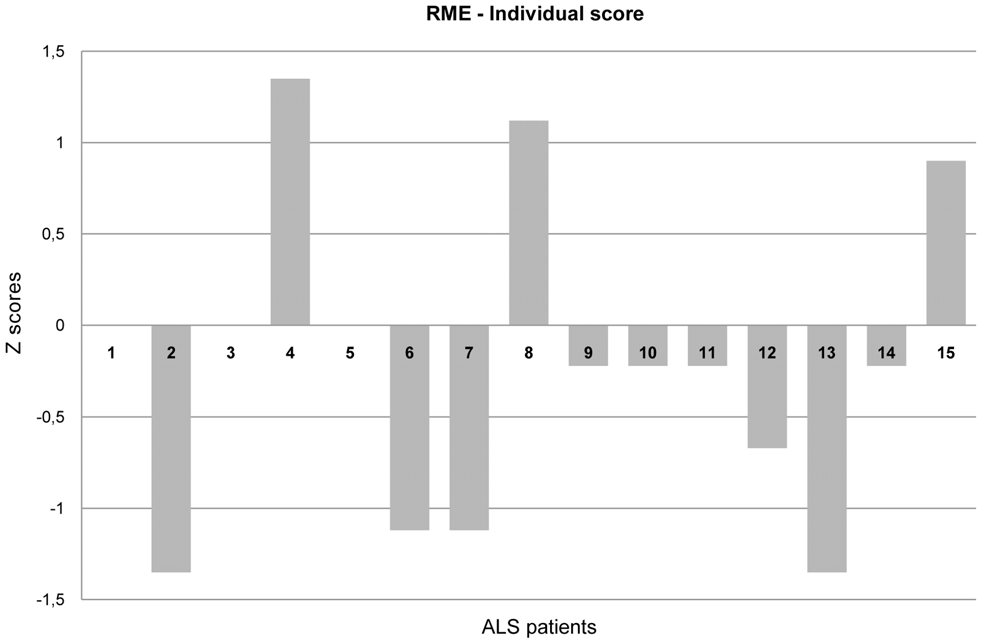
RME experimental test: Individual patients' scores. RME Reading the Mind in the Eyes.

Comparison of individual patient scores with healthy controls' mean [51] for the RME task did not show the presence of any significant differences between patients' and healthy controls' scores. Regarding the RME control task, patients' performance was equivalent to healthy controls' performance. Individual patient scores for the experimental task, expressed as Z scores, are shown in Figure 2.

**Figure 3:**
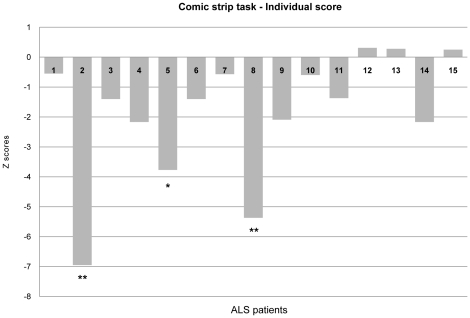
Story completion task: Individual patients' differences (SC–N-SC). N-SC Non-Social items; SC Social items. * p<0.05; ** p<0.01.

To investigate whether the patients' performance on the SC items was worse than their performance on the N-SC items, individual patients' difference scores between SC and N-SC stories (i.e. SC score–N-SC score) were compared with healthy controls by means of the revised standardised difference test [53]. The comparison showed the presence of a difference in 12 out of 15 patients (80.00%) in the direction stated by the experimental hypothesis (SC<N-SC) and this difference was statistically significant in patients 2 (t(21) 4.962, p<0.001), 5 (t(21) 2.607, p<0.05) and 8 (t(21) 3.784, p<0.001). Individual patients' differences (SC–N-SC), expressed as Z scores, are shown in Figure 3.

## Discussion

The present study was aimed at investigating a specific ToM ability, i.e. the ability of correctly interpreting social situations by attributing intentions to others appropriately, and demonstrated that ALS patients showed a specific deficit in understanding social intentions. To the best of our knowledge this is the first study which has examined and compared the understanding of social and non-social contexts in patients with ALS and shown a specific and selective deficit in the former only. This deficit is parallel to that one found in a previous study of a small group of FTD patients [33]. The current finding further supports the notion of a continuum of cognitive dysfunction ranging from ALS to FTD, with parallel cognitive profiles in both disorders.

The pattern of results followed the experimental hypothesis: more precisely, ALS patients' performance on the non-social stories was significantly better than their performance on the social stories. This pattern of results was not found in healthy controls. Furthermore, when the performance of the two groups was directly compared, the performance of patients with ALS and healthy controls significantly differed on the social element only with an impairment in patients. Single case analysis confirmed the previous findings at an individual level, with 12 of the 15 patients showing the same direction of effect and three with clearly abnormal performance significantly different from controls. However, one caveat of this study is that the performance of the healthy controls is approaching ceiling on both social and non-social items, as is the performance of the ALS patients on the non-social items. Therefore, it is possible that the social stories are relatively more difficult than the non-social ones and future work should address whether this is the case. The analysis of reaction times revealed that even if patients with ALS were typically slower than healthy controls throughout the task, the reaction times associated with the execution of the non-social and social dimensions did not differ from each other within each group of participants.

The previous studies directly investigating ToM abilities in patients with ALS have provided heterogeneous results [25]–[27]. While ALS patients were not significantly impaired on tasks involving humorous cartoons and stories with mental and physical scenarios [25], they were impaired on the Judgement of Preference task, showed a trend towards significantly lower RME accuracy scores [26] and were impaired in their ability to understand behaviour in social situations using the Faux Pas task [27]. In this latter study Meier and colleagues [27] found a specific effect of the task, i.e., their ALS patients showed a poorer performance in stories containing social interactions that involve a faux pas than in the control stories in which the faux pas was removed. As in both the faux pas and the control stories a social interactions is actually involved, Meier et al. 's study is only partially in line with the present one, and thus we suggest that future research should aim at better understanding what features of a social interaction make it more difficult for ALS patients its comprehension (e.g., interaction involving affective rather than cognitive components of ToM, interaction that occurs at the present time rather than in the future, and so on).

The main difference between the present study and the previous ones in the literature is the focus we place on the ability to comprehend social and non-social situations. For example, Gibbons and colleagues [25] used a combination of social and non-social situations in the same experimental category, without a clear distinction between the presence or absence of social interactions. Our results may differ from those reported in the previous studies due to their failure to consider situations with both social and non-social contexts. Moreover, Gibbons and colleagues used humorous situations in both their mental and physical conditions and, as suggested by the authors, a general problem in reasoning and inferential capability contributed to their patients' performance on both the mental and physical tasks.

One of the most currently debated issues in cognitive neuroscience is whether the cognitive and neural processes involved in perception, language, memory, and attention, actually suffice to account for the ways in which we conduct our social interactions, or whether there are specific brain structures and cognitive mechanisms to cope with social complexity [54]–[56]. ToM represents one of the key everyday-life complex abilities of understanding and interpreting social situations in order to behave accordingly. Several studies in both the neuropsychological [57]–[59] and neuroimaging (see [60] for a review) literature reported the prefrontal cortices as a key brain region underlying ToM. In particular, Walter et al. [31] and Ciaramidaro et al. [29] via a series of fMRI experiments have demonstrated that the medial prefrontal cortices are involved in understanding the intentions of people involved in social interactions (e.g. social intentions) but not in understanding the intentions of people outside social interactions (e.g. private intentions). Dysfunction of this region in patients with ALS has been indicated by a range of neuroimaging studies [6]–[8].

It has been recently proposed that the social and behavioural problems that often characterize frontal neurodegenerative diseases such as FTD–i.e. alterations of patients' social behaviour and conduct in terms of disinhibition and loss of control or, conversely, apathy and loss of concern [61]–[62]–may at least partially be the result of a significant impairment in social understanding ability [20]–[21]. Similar although less severe behaviour abnormalities have been reported in ALS [4] with irritability and disinhibition [63] and apathy [64]. The deficit in social cognition reported here may underlie such changes.

Regarding the RME task, ALS patients' performance either as a group or as individuals did not differ from healthy controls' performance. According to the literature, the studies involving patients with FTD have almost invariably demonstrated an impaired performance on this task, with the exception of a single case study [65] which showed a good performance on it. One could argue that the discrepancy between FTD and ALS patients' performance on the RME task may depend on the fact that the stimuli that make up this task may require a 'cognitive integrity' to be analysed appropriately that is seriously compromised in FTD but not in ALS. According to our experimental hypothesis, we consider the good patients' performance on the RME as independent evidence of our prediction, as the RME is a ToM task not requiring the comprehension of social situations. Further evidence will be necessary to support this conclusion.

The neuropsychological assessment did not show the presence of significant differences between ALS patients' and healthy controls' performance on the vast majority of tasks: more precisely, only the performance on the verbal fluency tasks and the GNT were significantly different, with patients getting lower scores than healthy controls, in keeping with previous studies on cognitive impairment in ALS [2], [6]–[7]. Thus, the patients involved in the present study did not show the presence of marked cognitive impairment that could interfere negatively with their performance on the experimental tasks proposed.

The ALSFRS-R administered to each patients allowed us to identify the nature of the functional impairment at the time of testing, with approximately half of the patients (n 6) who presented with bulbar signs while the others (n 9) presented with upper and lower limb involvement. As it has been suggested that the presence of bulbar signs may be related to increased cognitive change by some studies [40], we compared patients with and without bulbar signs, although clearly the interpretation is limited by small sample size. Only the Verbal Fluency Letter task and the Verbal Fluency index showed a statistically significant difference (bulbar<non-bulbar), but the performance on the social stories of the ToM task was impaired in both subgroups relative to controls. Thus, overall results demonstrated the absence of significant differences in the cognitive and ToM abilities of the two subgroups of patients involved in the current study.

It should be noted that our paradigm was adapted from an experimental protocol previously administered in our series of fMRI studies [29]–[32], which considered physical interaction and private intention items as two examples of N-SC stories. These two kinds of items differ from one another as the latter involve a character, while the former do not. We used this protocol in spite of this limitation because one of the aims of our present study was to find convergent neuropsychological evidence in a group of patients thought to have prefrontal dysfunction with our neuroimaging findings which demonstrate the prefrontal cortex plays a crucial role in the comprehension of social situations. However, even if the comparison in the ALS group of the two different types of non-social stories resulted in a statistically significant difference, when the score of the private intentions items was compared with the averaged score on the social stories, a statistically significant difference still occurred, allowing us to rule out the possibility that the different nature of the physical interaction and private intention items might have played a significant role in determining the pattern of results of the present study. Patients were also assessed on the RME task, a task not requiring the comprehension of social contexts to determine whether ALS patients' poor performance is restricted to SC conditions. Future studies should investigate these findings further using characters in both physical interaction and private intention item.

In conclusion, our results provide the first evidence on the presence of specific deficits in the domain of social understanding in ALS patients, and support the notion of a link between FTD and ALS with parallel ToM deficits in both groups indicating subclinical levels of FTD in some non-demented ALS patients.
